# Perspective: IL-15 cytokine-armored NK cells as ready-to-use immunotherapy for diverse malignancies: therapeutic potential and toxicity risks

**DOI:** 10.3389/fimmu.2025.1704404

**Published:** 2025-11-19

**Authors:** Scott L. Baughan, Timothy Folsom, Matt Johnson, Joshua Kreuger, Beau R. Webber, Branden S. Moriarity

**Affiliations:** 1 Department of Medicine, University of Minnesota Twin Cities, Minneapolis, MN, United States; 2 Center for Genome Engineering, University of Minnesota, Minneapolis, MN, United States; 3 Department of Pediatrics, University of Minnesota, Minneapolis, MN, United States; 4 Masonic Cancer Center, University of Minnesota, Minneapolis, MN, United States

**Keywords:** natural killer (NK) cells, chimeric antigen receptors (CARs), CAR-NK cells, IL-15 cytokine-armored CAR-NK, cytokine release syndrome, CAR-T cell therapy, cytokine release syndrome (CRS)

## Abstract

Natural killer (NK) cells have been engineered to express chimeric antigen receptors (CARs) to enhance their cytotoxic capabilities through CAR-mediated activation, a strategy that has yielded promising advancements in cancer treatment in recent pre-clinical and clinical trials. However, the use of CAR-NK cells for the treatment of solid tumors has presented challenges due to limited *in vivo* CAR-NK efficacy, expansion, persistence, and the suppressive tumor microenvironment. Many groups have developed IL-15 cytokine-armored CAR-NK therapeutics targeting various cancers to overcome these challenges. However, preclinical *in vivo* studies using immunodeficient mice have encountered instances of significant toxicity without evidence of cytokine release syndrome. The lack of an intact immune system likely allows for unchecked *in vivo* expansion of cytokine armored CAR-NK cells, leading to early mortality in immunodeficient mice following treatment with these cells. We speculate that the use of humanized mice will allow for engraftment of tumor and alleviate cytokine armored CAR-NK toxicity, thereby allowing for effective assessment of CAR-NK efficacy in the absence of toxicity.

## Introduction

Just over two million new cancer cases and 600, 000 cancer related deaths are projected to occur in 2025 (1). In addition, the incidence of the top six types of cancer (breast, prostate, lung/bronchus, colon/rectum, urinary/bladder, and melanoma) is on the rise (1). Despite great advances in early diagnosis and treatment, including an array of highly effective immuno- and targeted therapies, there remains an urgent need for breakthrough treatments capable of addressing a variety of cancer types.

The development of chimeric antigen receptor (CAR) T cell therapy has been a crowning achievement of genetic engineering and immunotherapy (2). However, the widespread availability of CAR-T cell therapy has been limited by the requirement for autologous (patient derived) CAR-T cells to avoid graft versus host disease (GvHD), greatly increasing the cost and complexity of their manufacturing (2, 3). The extended period between T cell collection and deployment of autologous CAR-T cell products risks tumor progression, evolution, and relapse, especially in the case of rapidly progressing malignancies (2). An additional key challenge in the use of CAR cell therapy is the requirement for a tumor-specific surface antigen for CAR targeting, limiting its application to cancers with identifiable cancer associated surface markers not expressed on normal tissues. This requirement restricts therapeutic potential and has been implicated in tumor escape and evolution, leading to evasion and relapse (2, 4, 5). Furthermore, the risk of treatment related toxicities, including cytokine release syndrome (CRS) and immune effector cell-associated neurotoxicity syndrome (ICANS) continue to limit therapy with CAR-T cells, often requiring expensive additional treatment, including intensive care (6, 7).

At present, CAR-T cell therapies approved for use by the FDA are exclusively built on an autologous αβ T cell chassis, with the CAR delivered using a viral vector. While effective, this manufacturing model limits the scope of CAR-T cell therapy and results in high cost and long manufacturing timelines (7, 8). Moreover, CAR-T cell therapy for solid tumors has thus far shown limited efficacy, and all current generations of approved CAR-T cell therapies are for hematologic malignancies for which common tumor surface receptors have been easily identified, namely CD19 (7, 8).

## Toward the development of CAR-NK cell therapeutics

Efforts to address the aforementioned shortcomings of CAR-T cells have led to the development of CAR engineered NK (natural killer) cell therapies, which have several advantages over standard CAR-T cell therapies. NK cell cytotoxicity is mediated through a balance of activation and inhibitory molecules expressed by target cells. Activating receptors such as NKG2D, NKG2C, and CD16, will activate NK cells after encountering their cognate ligand on target cells (9, 10). Conversely, killer immunoglobulin-like receptors (KIRs), natural killer group 2 member A (NKG2A), T cell immunoglobulin and ITM domain (TIGIT), among others, act as inhibitory receptors to greatly reduce NK cell activation and cytotoxicity when bound by their cognate ligand expression on target cells (reviewed in (9)). Thus, NK cell activation and cytotoxicity is determined by a balance of the relative expression of inhibitory and activation receptor ligands by individual target cells, enabling NK cells to innately recognize and kill transformed or virally infected cells (11, 12). For example, many cancer cells upregulate the stress-induced ligands MHC class I polypeptide related sequence A and B (MICA and MICB), which bind to the activating receptor NKG2D, and downregulate MHC Class I, which are then unable to bind inhibitory receptors, such as KIRs, potentiating NK cell activation and target cell killing (13–15).

The manufacturing process for NK cell-based therapies differs considerably from the process for CAR-T cell therapies. As mentioned above, while CAR-T cell therapies must be individually produced for each patient, “off the shelf” allogenic NK cells for patient treatment are possible and may even be advantageous in CAR-NK cell therapy, as NK cells do not recognize self vs non-self through MHC antigens, and thus cannot cause graft versus host disease (16). NK cells for CAR-NK cell therapy can be derived from several sources: donor cells from stored cord blood, induced pluripotent stem cells (iPSC), immortalized cell lines such as NK-92, or directly from patient peripheral blood for autologous transfer (17). Furthermore, despite limited efficacy in initial clinical trials (18–20), NK cell therapies have yielded promising results in recent clinical trials against hematologic cancers (21, 22). Treatment with cord-blood derived HLA-mismatched anti-CD19 CAR-NK cells led to a 73% response rate in chronic lymphocytic leukemia (CLL) patients, while treatment with purified CD56(+)CD3(-) NK cells from haploidentical KIR-ligand-mismatched donors resulted in a 54% response rate in acute myeloid leukemia (AML) patients at various stages. In addition, both studies reported that the treatment was well tolerated, with dose limiting toxicity and major side effects not reached in any patient.

NK based therapeutics have also been applied in preclinical models and clinical trials for a variety of solid tumors, including prostate, ovarian, gastroesophageal, brain (glioblastoma), pancreatic, and hepatocellular cancers, but with limited efficacy (8, 19, 20). In preclinical *in vivo* solid tumor models, NK cell therapies have been mostly unsuccessful in complete tumor clearance or the induction of durable remission (16, 20). Some of the limited efficacy observed in solid tumors can be attributed to the problems shared between CAR-T and CAR-NK cells, including antigen selection for the CAR, tumor heterogeneity and evolution, tumor mediated endothelial changes compromising immune cell homing to the tumor, and a hostile tumor microenvironment (TME) limiting immune cell penetration into the tumor (8, 23–25).

The trafficking and infiltration of CAR-NK cells into solid tumors remain barriers that need to be overcome prior to successful clinical implementation of CAR-NK cell therapy. The physical structure of the tumor and the biochemical nature of the TME are frequently impervious to immune cells (26). Intratumoral trafficking of NK cells is mediated by the chemokine receptors CCR2, CCR5, CCR7, CXCR3, and CXCR1, which engage with intratumoral ligands enabling cell penetration (27). However, during tumor development, many tumors downregulate and/or modify surface receptor expression and the tumor endothelium, inhibiting immune cell intravasation into the tumor [ex: reduced CXCL9/10 expression within the tumor (28) or suppression of adhesion molecules such as VCAM-1 and ICAM-1 within the endothelium (29)] (30–36). In addition, the TME is profoundly immunosuppressive for immune cells that do successfully infiltrate the tumor (37). Many tumors secrete soluble factors including IL-4, TGF-β, adenosine, and PGE_2_, which downregulate activating receptors (e.g., NKG2D) and impair immune cell metabolism (38–40). Furthermore, the hypoxic and nutrient deficient conditions in the TME stunt immune cell activity and proliferation (41, 42). Finally, expression of ligands such as PD-L1, CD155, and HLA-E by the tumor and subsequent engagement of the corresponding receptors PD-1, TIGIT, and NKG2A on infiltrating immune cells suppresses immune cell function, leading to anergy (43–46). Enhanced cell engineering strategies have been pursued to overcome these challenges and enhance tumor homing. These have included overexpression of CXCR2, which improved tumor penetration in preclinical models (47). An alternative method successfully employed IL-4 switch receptors engineered to instead activate IL-15 intracellular signaling to overcome the suppressive nature of the TME on CAR-T, which could likewise be deployed in CAR-NK cells (48). Engineering strategies for cell-based therapeutics including increasing resistance to TGF-β signaling (49), knockout of inhibitory ligands (50), and cytokine armoring (see multiple studies in detail, below) or co-stimulation (51) have been employed with success in pre-clinical models. Further investigation and engineering to optimize chemokine receptor expression to match tumor chemokine profiles combined with optimized construction of cell therapies for resistance to the suppressive TME and enhanced function within it will be essential to further improving the efficacy of CAR-NK cell therapy for solid tumors.

However, NK cell dysfunction, exhaustion, and limited persistence are likely the primary reasons for their reduced efficacy (52). *In vivo*, NK cells have a short half-life, typically less than 2–3 weeks in preclinical models and clinical trials (53). Despite high efficacy *in vitro*, CAR-NK cells have been shown to rapidly lose function *in vivo* (16, 54–56).

Exhaustion, anergy, and senescence are naturally occurring phenomena which serve to prevent autoimmunity (57). This same function likewise prevents widespread and persistent activation of anti-tumor immune cells within the host. Exhaustion of NK cells is thought to be the result of receptor mediated inhibition, while anergy is the result of receptor activity in the absence of co-stimulation (52, 58). Both exhaustion and anergy are distinct mechanisms that result in a reversible impairment from which immune cells can be rescued to continue expansion and division, either via immuno-stimulation or by cytokine or small molecule stimulation (17, 59, 60). These modalities can restore the capacity of the NK cells to proliferate and exert cytotoxic effects on target cells. In contrast, senescence, which is the result of repeated clonal expansion and is characterized by epigenetic remodeling and telomere shortening, leads to more permanent arrest of cellular division, and is irreversible via cytokine or receptor stimulation (52). Anergy, exhaustion, and senescence likely limit the efficacy and persistence of adoptively transferred NK cells *in vivo.* Thus, overcoming these dimensions of NK cell dysfunction is a key focus of ongoing research for NK therapeutics.

Cytokine stimulation is a major avenue for revitalizing anergic and exhausted lymphocytes. In particular, the challenges of persistence and exhaustion have been addressed through the use of cytokine supplementation, primarily with IL-15 and IL-21. Interleukin-15 (IL-15) is a γc-family cytokine and is a key effector of development, homeostasis, and cytotoxic functions of NK cells (61). NK cells are generally exposed to IL-15 by *trans* presentation via dendritic cells or monocytes utilizing the IL-15Rβ surface molecule (CD122) (62). IL-15 stimulation activates JAK1/3 and downstream STAT5, as well as the PI3K–AKT–mTOR pathway, which together promote survival, proliferation, metabolic fitness, and resistance to exhaustion in NK cells (63). IL-15 signaling also enhances mitochondrial biogenesis and glycolytic capacity of NK cells, supporting sustained NK cell function within the otherwise metabolically constrained tumor microenvironment (63–65). Altogether, these effects make IL-15 stimulation an attractive method for enhancing the function of CAR-NK cell therapies *in vivo* [reviewed in (64, 66)]. In particular, using IL-15 cytokine armoring, persistence and cytotoxic function can be enhanced without the need of exogenous systemic cytokine treatment, potentially avoiding an array of side effects.

Many groups have developed IL-15 cytokine armored CAR-NK therapies targeting a variety of cancers in order to overcome these challenges but some have encountered toxicity in *in vivo* models (67). Stimulation of CAR-NK cells by IL-15 induces upregulation of the AKT serine threonine kinase 1 (AKT) and mammalian target of rapamycin (mTOR) pathways and the suppression of cytokine inducible SH2 containing protein (*CIS*) (67, 68). This in turn leads to an enhancement of the cellular proliferation capacity and resistance to exhaustion and anergy (68). Auto-secretion of IL-15, referred to as cytokine armoring, has been shown to be an effective means of overcoming the limited *in vitro* and *in vivo* expansion of cell based therapeutics, and multiple clinical trials have been completed or are in progress investigating IL-15 armored CAR-T and CAR-NK cell based therapies (67, 69, 70). Such autocrine enhancement overcomes the problem of external supplementation of IL-15 and the side effects thereof, and renders the CAR-NK cells largely self-sufficient.

## Pre-clinical studies of IL-15 cytokine armored CAR-NK cells: balancing toxicity and treatment

In multiple pre-clinical investigations, significant toxicity has been observed following treatment of immunodeficient animal models with CAR-NK cells constitutively expressing stimulatory cytokines. These are briefly reviewed below and in **Table 1**. CAR-NK cells derived from cord blood and engineered to express soluble IL-15 were observed to have significant toxicity in immunodeficient mice, especially at high doses (71). Here, a retrovirally delivered expression cassette containing anti-CD19-CAR, IL-15, and a inducible caspase 9 suicide gene expressed as a single mRNA was used to transduce cord blood derived NK cells, resulting in IL-15 secreting CAR-NK cells (termed iC9/CAR.19/IL15 NK cells) (methodology described in (75, 76)). The authors used NCG (NOD Prkdc Il2r Gamma) mice, which lack B, T, and NK cells and have improved durability of the immunodeficient phenotype with age compared with other NOD/SCID models. NCG mice, injected with 1x10^4^ mixed Raji (human Burkitt lymphoma) or Jeko-1 (human mantle cell lymphoma) cells on day 0 and treated with 1x10^7^ iC9/CAR.19/IL15 NK cells achieved an approximately 40% cure rate. However, early toxicity resulted in substantially increased mortality for mice injected with IL-15 expressing CAR-NK cells before the mice carrying tumor xenografts without treatment or mice treated with non-IL-15 expressing cells had begun to exhibit decreased survival. This toxicity was hypothesized to be the result of excessive cytokine (IL-15) release, but this was not confirmed experimentally.

**Table 1:** Pre-clinical studies of cytokine armored CAR-NK cell therapies.

Study	Cancer model	NK cell modification	Dosing & timing	Efficacy	Toxicity observed
Liu et al., 2018 (71)	CD19^-^ Raji & Jeko-1 (MCL) xenografts in NCG mice	CAR-NK cells secreting IL-15	1×10^7^ CAR-IL15 NK cells, 3 days post tumor	~40% cure rate	Early mortality attributed to cytokine toxicity
Christodoulou et al., 2021 (72)	M4-V-11 (AML) xenograft in NSG mice	CD28-CAR-NK secreting IL-15	1×10^7^ CAR-IL15 NK cells (standard protocol); or 3×10^6^ CAR-IL15 NK cells (modified protocol)	Improved tumor clearance	Median survival decreased; systemic toxicity
Van den Eynde et al., 2024 (30)	Raji (CD70^+^) xenograft in NSG mice	CD70-CAR NK with secretory IL-15 (via P2A)	1×10^7^ IL15 NK cells, post 10 Gy irradiation	Modest tumor control	Transient weight loss, otherwise well tolerated
Guo et al., 2024 (73)	CD19^-^ Raji xenografts in NSG mice	CD70-CAR NK with IL-15 (P2A cassette)	High dose: 2×10^5^ cells Modified: 1×10^5^ × 2 doses	4/5 mice achieved complete response (modified dosing)	Lethal systemic toxicity at high dose; resolved with split dosing
Shanley et al., 2024 (74)	glioblastoma xenograft in NSG mice	NK cells expressing either IL-15 or IL-21	1×10^5^ intratumoral NK cells, 7 days post tumor engraftment	IL-15 group showed early tumor control	IL-15: Early mortality, weight loss, reduced survival
Wang et al., 2025 (50)	Raji (PD-L1^+^, CD155^+^) xenografts in NSG mice	CD19-CAR NK with IL-15 and triple knockout (TPC: TIGIT, PDCD1, CISH)	2 × 5×10^6^ cells on days 3 and 17	Improved tumor control; prolonged survival	Rapid systemic toxicity, early loss of 3/5 mice

A second preclinical study investigated peripheral blood derived NK cells engineered to constitutively secrete IL-15 via transduction with a retroviral vector encoding an anti-CD123-CAR transcriptionally linked to secreted IL-15 (72). NOD SCID gamma (NSG) mice were injected with 1x10^6^ M4V-11 (human acute myeloid leukemia) cells and treated seven days later with either 1x10^7^ CAR-IL-15 NK cells or standard CAR-NK cells. Mice receiving CAR-IL-15 NK cells showed reduced overall survival (21 days post-tumor injection) compared to those treated with standard CAR-NK cells (25 days). A modified protocol was tested, comparing a single-dose therapy of 3x10^6^ CAR-IL-15 NK cells four days post-tumor injection to a three-dose regimen of 3x10^6^ standard CAR-NK cells on days four, seven, and ten. While the standard CAR-NK cell treatment significantly extended survival compared to untreated controls (78 days vs. 49 days), the CAR-IL-15 NK cell treatment markedly reduced survival (26 days vs. 49 days). Notably, mice treated with IL-15-secreting NK cells lacking the CAR also exhibited reduced survival (21 days vs. 49 days), suggesting that the decreased survival likely stemmed from IL-15-related cytokine toxicity.

In an alternative approach building from a NK cell line, NK-92, anti-CD70 CAR-NK cells which constitutively express and secrete IL-15 from a similarly designed cassette resulted in less lethality to the mouse models but showed only mild efficacy in tumor cytotoxicity (77). The study authors initially found that IL-15 supplementation markedly increased the density of anti-CD70 CAR expression on NK cells when co-cultured with cancer cells, thus the IL-15 expression cassette was included downstream of the CAR in the plasmid expression vector, resulting in secretion of the cytokine. The expression vector was introduced to the NK-92 cells via electroporation. NSG mice were injected with 1x10^6^ Raji cells and subsequently treated with 1x10^7^ CAR-NK cells zero- and four-days post tumor xenograft. In contrast to other examples, the CAR-NK cells had been treated with 10 Gy of radiation four hours post electroporation of the CAR construct, prior to injection into the mice. This strategy prevented toxicity beyond transient weight loss in all three treatment groups of mice (Mock, CD70 CAR, and CD70 CAR-IL15). However, the median survival time for the mice treated with the CAR-IL-15 NK therapy was only 27 days vs 21.5 for the other groups, a modest difference. We speculate that this is due to the fact that the irradiated NK cells were unable to undergo expansion *in vivo* and therefore could not effectively kill the xenograft tumor faster than the tumor cells were able to proliferate. Further investigation into whether or not this limitation could be overcome by using multiple subsequent injections may be beneficial.

Another anti-CD70 CAR-NK cell product evaluated against models of B-Cell lymphoma demonstrated efficacy but resulted in lethal toxicity at high doses (73). Here, the authors treated mice with NK cells derived from the NK92MI cell line carrying an anti-CD70-CAR-IL15 expression cassette introduced via lentiviral transduction. Mice were initially treated with 2x10^5^ IL-15 armored CAR-NK cells three days post seeding of 1x10^4^ mixed CD19+ and CD19 knockout Raji tumor cells. However, all mice died within two weeks of treatment, which the authors attribute to systemic toxicity from the inflammatory response of the CAR-NK cells. A modified protocol was conducted in which the mice were seeded with the same dose of tumor cells and then treated with 1x10^5^ CAR-NK cells with a second identical dose five days later for a total 20:1 effector to initial target dose. This resulted in complete tumor eradication in four of five mice, and median survival was not reached within the study period of 113 days. Similarly constructed CD19-CAR-IL15 cells failed to eradicate tumors in this mouse model, achieving only a modest increase in survival time (34 days vs 30 for controls).

In xenograft mouse models of glioblastoma, administration of IL-15 armored NK cells without a CAR resulted in rapid lethality compared to IL-21 cytokine armoring (74). Here, umbilical cord blood derived NK cells were engineered to express either IL-21 or IL-15 from a retroviral vector. 5x10^5^ patient derived xenograft glioblastoma cells were intracranially implanted into NSG mice. Seven days post tumor seeding, 1x10^5^ IL-15, IL-21, or non-transduced (NT) NK cells were injected intratumorally. In mice treated with IL-15 NK cells, overall survival was markedly reduced compared to untreated controls and NT NK cells treated mice (median survival 37 days vs > 70 days for non-transduced and untreated controls; exact numbers were not reported). Mice treated with IL-15 secreting NK cells additionally displayed significant early weight loss, signaling potential increased systemic toxicity. While this study did not employ CAR-NK cells, it is useful for comparison as it likewise demonstrates signs of systemic toxicity with IL-15 cytokine armoring.

Finally, rapid onset systemic toxicity, presenting as weight loss, and organ swelling, was also observed alongside drastic decrease in tumor volume in an *in vivo* assessment of IL-15 armored anti-CD-19 CAR-NK cells by our group (50). Here, CAR-NK cells derived from peripheral blood were modified using CRISPR base editing to remove combinations of intra and extra cellular inhibitory signals aryl hydrocarbon receptor (*AHR)*, cytokine-inducible SH2-containing (*CISH*), programmed death containing domain 1 (*PDCD1*)*, TIGIT*, and killer cell lectin-like receptor 1 (*KLRG1*). When challenged against Raji tumor cells *in vitro*, these CAR-NK cells exhibited markedly increased killing efficacy with triple knockout of *TIGIT, PDC1*, and *CISH* (TPC) showing the highest efficacy. An anti-CD19-CAR-T2A-RQR8-P2A-IL-15 expression construct was delivered to these NK cells using the *TcBuster* transposon system contemporaneously with base editing. NSG mice were injected with 1x10^5^ Raji cells overexpressing PD-L1 and CD155 and treated with two doses of 5x10^6^ CAR-NK or CAR-NK TPC cells at days three and 17. While the treatment with IL-15 cytokine armored CAR-NK and CAR-NK TPC cells resulted in a marked increase in median survival (CAR-NK: 63 days vs CAR-NK TPC: 30 days vs control: 25 days), the median survival of the mice treated with CAR-NK TPC in particular was hampered by early systemic toxicity leading to the relatively early loss of three of the five mice that received this treatment. Weekly peripheral blood monitoring of these mice revealed that the CAR-NK TPC cells underwent rapid *in vivo* expansion shortly before systemic toxicity was observed. While IL-15 cytokine armored CAR-NK TPC treated mice exhibited improved tumor control, three mice died relatively early in the study from systemic toxicity. Thus, although overall survival was slightly improved compared to the CD-19 IL-15 armored CAR-NK cells alone, the recurring toxicity is indicative of a problem that must be overcome prior to the initiation of human trials with IL-15 armored CAR-NK TPC cells, similar to other studies mentioned in this section. Notably, in unpublished data from our group, these animals were assessed for heightened levels of murine IL-6 (CRS inducer) in serum and found to have similar levels as controls. Moreover, immunohistochemistry analysis of liver, lungs, and spleen identified massive infiltration of CD56+ NK cells.

## Closing remarks

As all these studies have employed immunodeficient mice as the test model, it is possible that the observed toxicity is related to the use of immunodeficient mice, which lack an intact immune system. Without a host immune response to the allogenic NK cells, the proliferation and cytokine release from the modified NK cells is unchecked, potentially leading to the observed toxicity.

**Figure 1:**
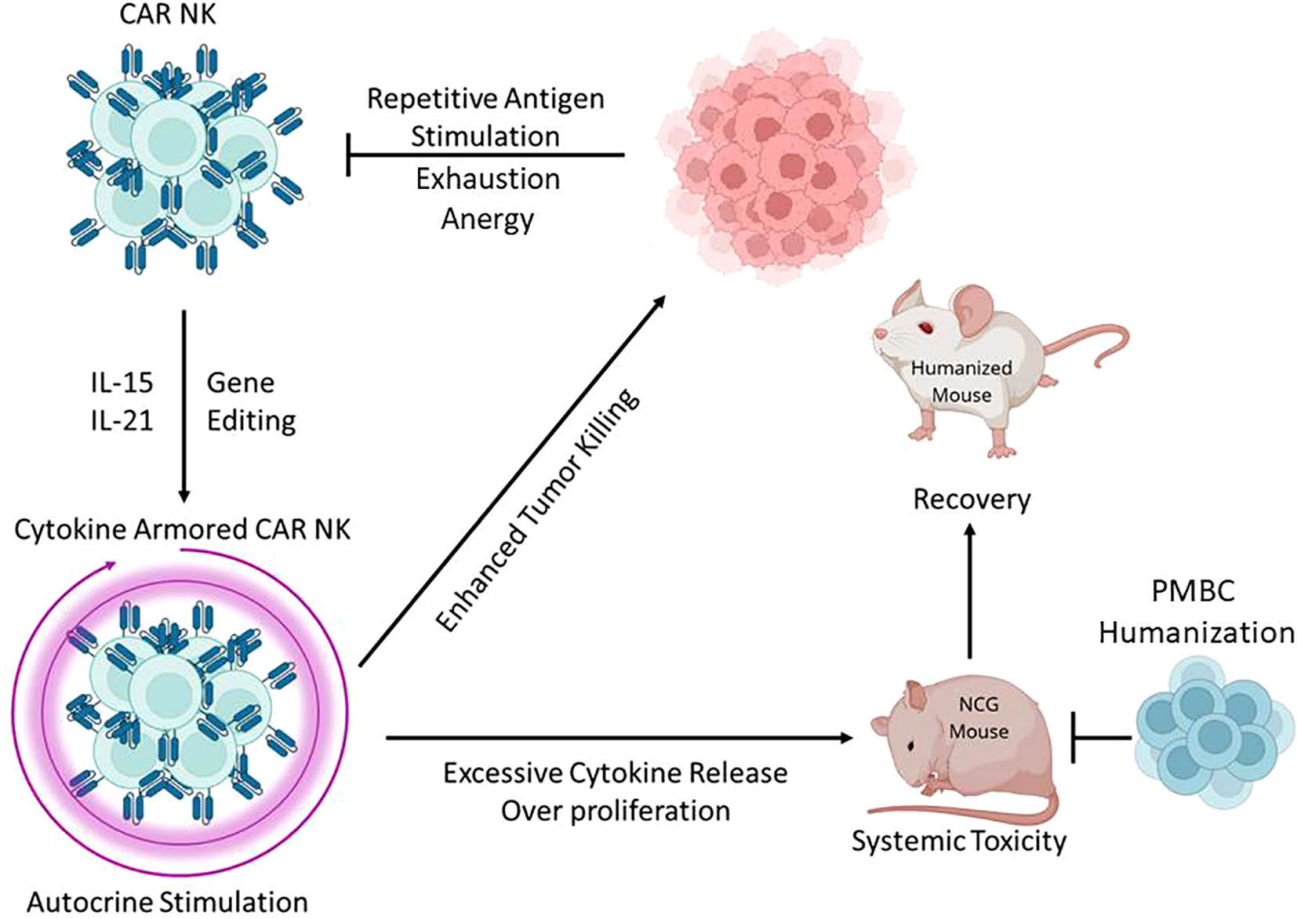
Repetitive antigenic stimulation from cancer cells and a non-permissive tumor microenvironment can lead to exhaustion, anergy, and senescence of CAR-NK cells. This has been overcome by cytokine armoring with expression of stimulatory molecules such as IL-15 and IL-21, as well as with gene editing to disable inhibitory signals. However, while this enhances the anti-tumor effect of CAR-NK cells, the excessive cytokine release and over proliferation has been toxic in immunodeficient mice. We hypothesize that the presence of an immune system would abrogate this excessive response, reducing the toxicity of the CAR-NK cell treatment and enabling recovery in preclinical animal models.

It is possible that once this therapy is tested in human patients possessing functional immune systems, the toxicity of the allogenic cytokine armored CAR-NK cells will be abrogated by the host’s immune response to the therapy itself (**Figure 1**). Additionally, these native immune cells may act as a cytokine sink for IL-15, reducing the otherwise toxic side effects of cytokine release during NK cell expansion. Finally, CAR-NK cells could be engineered with integrated, drug induced kill switches to prevent the unchecked expansion and toxicity as seen in mice. Together, these strategies would allow for successive waves of injection-expansion-cessation of the CAR-NK cell therapy, maximizing anti-tumor effect. In addition to the other benefits, the benevolent, limited side effect profile of allogenic CAR-NK cell treatment may enable the use of these in place of cytotoxic chemotherapy in frail patients who cannot tolerate such intensive therapies. It also presents the opportunity for the creation of “off the shelf” therapeutic options for the treatment of various cancers, including difficult to treat solid tumor malignancies.

## Data Availability

The original contributions presented in the study are included in the article/supplementary material. Further inquiries can be directed to the corresponding author.

## References

[1] Siegel RL Kratzer TB Giaquinto AN Sung H Jemal A . Cancer statistics, 2025. CA Cancer J Clin. (2025) 75:10–45. doi: 10.3322/caac.21871, PMID: 39817679 PMC11745215

[2] Brudno JN Maus MV Hinrichs CS . CAR T cells and T-cell therapies for cancer: A translational science review. Jama. (2024) 332:1924–35. doi: 10.1001/jama.2024.19462, PMID: 39495525 PMC11808657

[3] Cliff ERS Kelkar AH Russler-Germain DA Tessema FA Raymakers AJN Feldman WB . High cost of chimeric antigen receptor T-cells: challenges and solutions. Am Soc Clin Oncol Educ Book. (2023) 2023:e397912., PMID: 37433102 10.1200/EDBK_397912

[4] Abbott RC Cross RS Jenkins MR . Finding the keys to the CAR: identifying novel target antigens for T cell redirection immunotherapies. Int J Mol Sci. (2020) 21. doi: 10.3390/ijms21020515, PMID: 31947597 PMC7014258

[5] Majzner RG Mackall CL . Tumor antigen escape from CAR T-cell therapy. Cancer Discov. (2018) 8:1219–26. doi: 10.1158/2159-8290.CD-18-0442, PMID: 30135176

[6] Bonifant CL Jackson HJ Brentjens RJ Curran KJ . Toxicity and management in CAR T-cell therapy. Mol Ther Oncolytics. (2016) 3:16011. doi: 10.1038/mto.2016.11, PMID: 27626062 PMC5008265

[7] Khan SH Choi Y Veena M Lee JK Shin DS . Advances in CAR T cell therapy: antigen selection, modifications, and current trials for solid tumors. Front Immunol. (2024) 15:1489827. doi: 10.3389/fimmu.2024.1489827, PMID: 39835140 PMC11743624

[8] Pan K Farrukh H Chittepu V Xu H Pan CX Zhu Z . CAR race to cancer immunotherapy: from CAR T, CAR NK to CAR macrophage therapy. J Exp Clin Cancer Res. (2022) 41:119. doi: 10.1186/s13046-022-02327-z, PMID: 35361234 PMC8969382

[9] Voskoboinik I Whisstock JC Trapani JA . Perforin and granzymes: function, dysfunction and human pathology. Nat Rev Immunol. (2015) 15:388–400. doi: 10.1038/nri3839, PMID: 25998963

[10] Raulet DH Vance RE . Self-tolerance of natural killer cells. Nat Rev Immunol. (2006) 6:520–31. doi: 10.1038/nri1863, PMID: 16799471

[11] Yu Y . The function of NK cells in tumor metastasis and NK cell-based immunotherapy. Cancers. (2023) 15:2323. doi: 10.3390/cancers15082323, PMID: 37190251 PMC10136863

[12] Dunn GP Old LJ Schreiber RD . The immunobiology of cancer immunosurveillance and immunoediting. Immunity. (2004) 21:137–48. doi: 10.1016/j.immuni.2004.07.017, PMID: 15308095

[13] Koch J Steinle A Watzl C Mandelboim O . Activating natural cytotoxicity receptors of natural killer cells in cancer and infection. Trends Immunol. (2013) 34:182–91. doi: 10.1016/j.it.2013.01.003, PMID: 23414611

[14] Ascierto ML Idowu MO Zhao Y Khalak H Payne KK Wang X-Y . Molecular signatures mostly associated with NK cells are predictive of relapse free survival in breast cancer patients. J Trans Med. (2013) 11:1–11. doi: 10.1186/1479-5876-11-145, PMID: 23758773 PMC3694475

[15] Halfteck GG Elboim M Gur C Achdout H Ghadially H Mandelboim O . Enhanced *in vivo* growth of lymphoma tumors in the absence of the NK-activating receptor NKp46/NCR1. J Immunol. (2009) 182:2221–30. doi: 10.4049/jimmunol.0801878, PMID: 19201876

[16] Suck G Odendahl M Nowakowska P Seidl C Wels WS Klingemann HG . NK-92: an 'off-the-shelf therapeutic' for adoptive natural killer cell-based cancer immunotherapy. Cancer Immunol Immunother. (2016) 65:485–92. doi: 10.1007/s00262-015-1761-x, PMID: 26559813 PMC11029582

[17] Daher M Rezvani K . Next generation natural killer cells for cancer immunotherapy: the promise of genetic engineering. Curr Opin Immunol. (2018) 51:146–53. doi: 10.1016/j.coi.2018.03.013, PMID: 29605760 PMC6140331

[18] Hu W Wang G Huang D Sui M Xu Y . Cancer immunotherapy based on natural killer cells: current progress and new opportunities. Front Immunol. (2019) 10:1205. doi: 10.3389/fimmu.2019.01205, PMID: 31214177 PMC6554437

[19] Suen WC Lee WY Leung KT Pan XH Li G . Natural killer cell-based cancer immunotherapy: A review on 10 years completed clinical trials. Cancer Invest. (2018) 36:431–57. doi: 10.1080/07357907.2018.1515315, PMID: 30325244

[20] Miller JS Lanier LL . Natural killer cells in cancer immunotherapy. Annu Rev Cancer Biol. (2019) 3:77–103. doi: 10.1146/annurev-cancerbio-030518-055653

[21] Liu E Marin D Banerjee P Macapinlac HA Thompson P Basar R . Use of CAR-transduced natural killer cells in CD19-positive lymphoid tumors. N Engl J Med. (2020) 382:545–53. doi: 10.1056/NEJMoa1910607, PMID: 32023374 PMC7101242

[22] Curti A Ruggeri L D'Addio A Bontadini A Dan E Motta MR . Successful transfer of alloreactive haploidentical KIR ligand-mismatched natural killer cells after infusion in elderly high risk acute myeloid leukemia patients. Blood. (2011) 118:3273–9. doi: 10.1182/blood-2011-01-329508, PMID: 21791425

[23] Yan T Zhu L Chen J . Current advances and challenges in CAR T-Cell therapy for solid tumors: tumor-associated antigens and the tumor microenvironment. Exp Hematol Oncol. (2023) 12:14. doi: 10.1186/s40164-023-00373-7, PMID: 36707873 PMC9883880

[24] Smolarska A Kokoszka Z Naliwajko M Strupczewska J Tondera J Wiater M . Cell-based therapies for solid tumors: challenges and advances. Int J Mol Sci. (2025) 26:5524. doi: 10.3390/ijms26125524, PMID: 40564987 PMC12193280

[25] Peng L Sferruzza G Yang L Zhou L Chen S . CAR-T and CAR-NK as cellular cancer immunotherapy for solid tumors. Cell Mol Immunol. (2024) 21:1089–108. doi: 10.1038/s41423-024-01207-0, PMID: 39134804 PMC11442786

[26] Labani-Motlagh A Ashja-Mahdavi M Loskog A . The tumor microenvironment: A milieu hindering and obstructing antitumor immune responses. Front Immunol. (2020) 11. doi: 10.3389/fimmu.2020.00940, PMID: 32499786 PMC7243284

[27] Yao X Matosevic S . Chemokine networks modulating natural killer cell trafficking to solid tumors. Cytokine Growth Factor Rev. (2021) 59:36–45. doi: 10.1016/j.cytogfr.2020.12.003, PMID: 33495094

[28] Mikucki ME Fisher DT Matsuzaki J Skitzki JJ Gaulin NB Muhitch JB . Non-redundant requirement for CXCR3 signalling during tumoricidal T-cell trafficking across tumour vascular checkpoints. Nat Commun. (2015) 6:7458. doi: 10.1038/ncomms8458, PMID: 26109379 PMC4605273

[29] Griffioen AW Damen CA Martinotti S Blijham GH Groenewegen G . Endothelial intercellular adhesion molecule-1 expression is suppressed in human Malignancies: the role of angiogenic factors. Cancer Res. (1996) 56:1111–17., PMID: 8640769

[30] Tokunaga R Zhang W Naseem M Puccini A Berger MD Soni S . CXCL9, CXCL10, CXCL11/CXCR3 axis for immune activation - A target for novel cancer therapy. Cancer Treat Rev. (2018) 63:40–7. doi: 10.1016/j.ctrv.2017.11.007, PMID: 29207310 PMC5801162

[31] Pan M Wei X Xiang X Liu Y Zhou Q Yang W . Targeting CXCL9/10/11–CXCR3 axis: an important component of tumor-promoting and antitumor immunity. Clin Trans Oncol. (2023) 25:2306–20. doi: 10.1007/s12094-023-03126-4, PMID: 37076663

[32] Tokunaga R Zhang W Naseem M Puccini A Berger MD Soni S . CXCL9, CXCL10, CXCL11/CXCR3 axis for immune activation – A target for novel cancer therapy. Cancer Treat Rev. (2018) 63:40–7. doi: 10.1016/j.ctrv.2017.11.007, PMID: 29207310 PMC5801162

[33] Melssen MM Sheybani ND Leick KM Slingluff CL Jr . Barriers to immune cell infiltration in tumors. J Immunother Cancer. (2023) 11. doi: 10.1136/jitc-2022-006401, PMID: 37072352 PMC10124321

[34] Motz GT Santoro SP Wang LP Garrabrant T Lastra RR Hagemann IS . Tumor endothelium FasL establishes a selective immune barrier promoting tolerance in tumors. Nat Med. (2014) 20:607–15. doi: 10.1038/nm.3541, PMID: 24793239 PMC4060245

[35] Dirkx AE oude Egbrink MG Castermans K van der Schaft DW Thijssen VL Dings RP . Anti-angiogenesis therapy can overcome endothelial cell anergy and promote leukocyte-endothelium interactions and infiltration in tumors. FASEB J. (2006) 20:621–30. doi: 10.1096/fj.05-4493com, PMID: 16581970

[36] Bernardini G Gismondi A Santoni A . Chemokines and NK cells: regulators of development, trafficking and functions. Immunol Lett. (2012) 145:39–46. doi: 10.1016/j.imlet.2012.04.014, PMID: 22698182 PMC7112821

[37] Jia H Yang H Xiong H Luo KQ . NK cell exhaustion in the tumor microenvironment. Front Immunol. (2023) 14. doi: 10.3389/fimmu.2023.1303605, PMID: 38022646 PMC10653587

[38] Li H Han Y Guo Q Zhang M Cao X . Cancer-expanded myeloid-derived suppressor cells induce anergy of NK cells through membrane-bound TGF-beta 1. J Immunol. (2009) 182:240–9. doi: 10.4049/jimmunol.182.1.240, PMID: 19109155

[39] Lazarova M Steinle A . Impairment of NKG2D-mediated tumor immunity by TGF-β. Front Immunol. (2019) 10. doi: 10.3389/fimmu.2019.02689, PMID: 31803194 PMC6873348

[40] Vijayan D Young A Teng MWL Smyth MJ . Targeting immunosuppressive adenosine in cancer. Nat Rev Cancer. (2017) 17:709–24. doi: 10.1038/nrc.2017.86, PMID: 29059149

[41] Chambers AM Matosevic S . Immunometabolic dysfunction of natural killer cells mediated by the hypoxia-CD73 axis in solid tumors. Front Mol Biosci. (2019) 6:60. doi: 10.3389/fmolb.2019.00060, PMID: 31396523 PMC6668567

[42] Hatfield SM Kjaergaard J Lukashev D Schreiber TH Belikoff B Abbott R . Immunological mechanisms of the antitumor effects of supplemental oxygenation. Sci Transl Med. (2015) 7:277ra30. doi: 10.1126/scitranslmed.aaa1260, PMID: 25739764 PMC4641038

[43] Zhang Q Bi J Zheng X Chen Y Wang H Wu W . Blockade of the checkpoint receptor TIGIT prevents NK cell exhaustion and elicits potent anti-tumor immunity. Nat Immunol. (2018) 19:723–32. doi: 10.1038/s41590-018-0132-0, PMID: 29915296

[44] Beldi-Ferchiou A Lambert M Dogniaux S Vély F Vivier E Olive D . PD-1 mediates functional exhaustion of activated NK cells in patients with Kaposi sarcoma. Oncotarget. (2016) 7:72961–77. doi: 10.18632/oncotarget.12150, PMID: 27662664 PMC5341956

[45] Stanietsky N Simic H Arapovic J Toporik A Levy O Novik Av . The interaction of TIGIT with PVR and PVRL2 inhibits human NK cell cytotoxicity. Proc Natl Acad Sci U.S.A. (2009) 106:17858–63., PMID: 19815499 10.1073/pnas.0903474106 PMC2764881

[46] André P Denis C Soulas C Bourbon-Caillet C Lopez J Arnoux T . Anti-NKG2A mAb is a checkpoint inhibitor that promotes anti-tumor immunity by unleashing both T and NK cells. Cell. (2018) 175:1731–1743.e13. doi: 10.1016/j.cell.2018.10.014, PMID: 30503213 PMC6292840

[47] Kremer V Ligtenberg MA Zendehdel R Seitz C Duivenvoorden A Wennerberg E . Genetic engineering of human NK cells to express CXCR2 improves migration to renal cell carcinoma. J Immunother Cancer. (2017) 5:73. doi: 10.1186/s40425-017-0275-9, PMID: 28923105 PMC5604543

[48] Zhou Y Farooq MA Ajmal I He C Gao Y Guo D . Co-expression of IL-4/IL-15-based inverted cytokine receptor in CAR-T cells overcomes IL-4 signaling in immunosuppressive pancreatic tumor microenvironment. BioMed Pharmacother. (2023) 168:115740. doi: 10.1016/j.biopha.2023.115740, PMID: 37865999

[49] Burga RA Yvon E Chorvinsky E Fernandes R Cruz CRY Bollard CM . Engineering the TGFβ Receptor to enhance the therapeutic potential of natural killer cells as an immunotherapy for neuroblastoma. Clin Cancer Res. (2019) 25:4400–12. doi: 10.1158/1078-0432.CCR-18-3183, PMID: 31010834 PMC6635028

[50] Wang M Krueger JB Gilkey AK Stelljes EM Kluesner MG Pomeroy EJ . Precision enhancement of CAR-NK cells through non-viral engineering and highly multiplexed base editing. J Immunother Cancer. (2025) 13. doi: 10.1136/jitc-2024-009560, PMID: 40341025 PMC12067936

[51] Harrison AJ Du X von Scheidt B Kershaw MH Slaney CY . Enhancing co-stimulation of CAR T cells to improve treatment outcomes in solid cancers. Immunother Adv. (2021) 1:ltab016. doi: 10.1093/immadv/ltab016, PMID: 35919743 PMC9327106

[52] Judge SJ Murphy WJ Canter RJ . Characterizing the dysfunctional NK cell: assessing the clinical relevance of exhaustion, anergy, and senescence. Front Cell Infect Microbiol. (2020) 10:49. doi: 10.3389/fcimb.2020.00049, PMID: 32117816 PMC7031155

[53] Zhang Y Wallace DL de Lara CM Ghattas H Asquith B Worth A . *In vivo* kinetics of human natural killer cells: the effects of ageing and acute and chronic viral infection. Immunology. (2007) 121:258–65. doi: 10.1111/j.1365-2567.2007.02573.x, PMID: 17346281 PMC2265941

[54] Klingemann H Boissel L Toneguzzo F . Natural killer cells for immunotherapy - advantages of the NK-92 cell line over blood NK cells. Front Immunol. (2016) 7:91. doi: 10.3389/fimmu.2016.00091, PMID: 27014270 PMC4789404

[55] Tonn T Schwabe D Klingemann HG Becker S Esser R Koehl U . Treatment of patients with advanced cancer with the natural killer cell line NK-92. Cytotherapy. (2013) 15:1563–70. doi: 10.1016/j.jcyt.2013.06.017, PMID: 24094496

[56] Arai S Meagher R Swearingen M Myint H Rich E Martinson J . Infusion of the allogeneic cell line NK-92 in patients with advanced renal cell cancer or melanoma: a phase I trial. Cytotherapy. (2008) 10:625–32. doi: 10.1080/14653240802301872, PMID: 18836917

[57] Blank CU Haining WN Held W Hogan PG Kallies A Lugli E . Defining 'T cell exhaustion'. Nat Rev Immunol. (2019) 19:665–74. doi: 10.1038/s41577-019-0221-9, PMID: 31570879 PMC7286441

[58] Bald T Krummel MF Smyth MJ Barry KC . The NK cell-cancer cycle: advances and new challenges in NK cell-based immunotherapies. Nat Immunol. (2020) 21:835–47. doi: 10.1038/s41590-020-0728-z, PMID: 32690952 PMC8406687

[59] Cichocki F Grzywacz B Miller JS . Human NK cell development: one road or many? Front Immunol. (2019) 10. 10.3389/fimmu.2019.02078 PMC6727427 31555287

[60] Blank CU Haining WN Held W Hogan PG Kallies A Lugli E . Defining ‘T cell exhaustion’. Nat Rev Immunol. (2019) 19:665–74. doi: 10.1038/s41577-019-0221-9, PMID: 31570879 PMC7286441

[61] Waldmann TA . IL-15 in the life and death of lymphocytes: immunotherapeutic implications. Trends Mol Med. (2003) 9:517–21. doi: 10.1016/j.molmed.2003.10.005, PMID: 14659465

[62] Huntington ND Legrand N Alves NL Jaron B Weijer K Plet A . IL-15 trans-presentation promotes human NK cell development and differentiation *in vivo*. J Exp Med. (2009) 206:25–34. doi: 10.1084/jem.20082013, PMID: 19103877 PMC2626663

[63] Marçais A Cherfils-Vicini J Viant C Degouve S Viel S Fenis A . The metabolic checkpoint kinase mTOR is essential for IL-15 signaling during the development and activation of NK cells. Nat Immunol. (2014) 15:749–57. doi: 10.1038/ni.2936, PMID: 24973821 PMC4110708

[64] Keppel MP Saucier N Mah AY Vogel TP Cooper MA . Activation-specific metabolic requirements for NK cell IFN-γ Production. J Immunol. (2015) 194:1954–62. doi: 10.4049/jimmunol.1402099, PMID: 25595780 PMC4323953

[65] Dean I Lee CYC Tuong ZK Li Z Tibbitt CA Willis C . Rapid functional impairment of natural killer cells following tumor entry limits anti-tumor immunity. Nat Commun. (2024) 15:683. doi: 10.1038/s41467-024-44789-z, PMID: 38267402 PMC10808449

[66] Ma S Caligiuri MA Yu J . Harnessing IL-15 signaling to potentiate NK cell-mediated cancer immunotherapy. Trends Immunol. (2022) 43:833–47. doi: 10.1016/j.it.2022.08.004, PMID: 36058806 PMC9612852

[67] Zhou Y Husman T Cen X Tsao T Brown J Bajpai A . Interleukin 15 in cell-based cancer immunotherapy. Int J Mol Sci. (2022) 23. doi: 10.3390/ijms23137311, PMID: 35806311 PMC9266896

[68] Daher M Basar R Gokdemir E Baran N Uprety N Nunez Cortes AK . Targeting a cytokine checkpoint enhances the fitness of armored cord blood CAR-NK cells. Blood. (2021) 137:624–36. doi: 10.1182/blood.2020007748, PMID: 32902645 PMC7869185

[69] Nguyen R Doubrovina E Mousset CM Jin BY Okada R Zhang X . Cooperative armoring of CAR and TCR T cells by T cell-restricted IL15 and IL21 universally enhances solid tumor efficacy. Clin Cancer Res. (2024) 30:1555–66. doi: 10.1158/1078-0432.CCR-23-1872, PMID: 37910044 PMC11018485

[70] Chen S Yang L Xia B Zhu H Piao Z Jounaidi Y . A self-activating IL-15 chimeric cytokine receptor to empower cancer immunotherapy. Immunotargets Ther. (2024) 13:513–24. doi: 10.2147/ITT.S490498, PMID: 39403195 PMC11472742

[71] Liu E Tong Y Dotti G Shaim H Savoldo B Mukherjee M . Cord blood NK cells engineered to express IL-15 and a CD19-targeted CAR show long-term persistence and potent antitumor activity. Leukemia. (2018) 32:520–31. doi: 10.1038/leu.2017.226, PMID: 28725044 PMC6063081

[72] Christodoulou I Ho WJ Marple A Ravich JW Tam A Rahnama R . Engineering CAR-NK cells to secrete IL-15 sustains their anti-AML functionality but is associated with systemic toxicities. J Immunother Cancer. (2021) 9. doi: 10.1101/2021.09.23.461509, PMID: 34896980 PMC8655609

[73] Guo S Lei W Jin X Liu H Wang JQ Deng W . CD70-specific CAR NK cells expressing IL-15 for the treatment of CD19-negative B-cell Malignancy. Blood Adv. (2024) 8:2635–45. doi: 10.1182/bloodadvances.2023012202, PMID: 38564778 PMC11157212

[74] Shanley M Daher M Dou J Li S Basar R Rafei H . Interleukin-21 engineering enhances NK cell activity against glioblastoma via CEBPD. Cancer Cell. (2024) 42:1450–1466.e11. doi: 10.1016/j.ccell.2024.07.007, PMID: 39137729 PMC11370652

[75] Hoyos V Savoldo B Quintarelli C Mahendravada A Zhang M Vera J . Engineering CD19-specific T lymphocytes with interleukin-15 and a suicide gene to enhance their anti-lymphoma/leukemia effects and safety. Leukemia. (2010) 24:1160–70. doi: 10.1038/leu.2010.75, PMID: 20428207 PMC2888148

[76] Quintarelli C Vera JF Savoldo B Giordano Attianese GM Pule M Foster AE . Co-expression of cytokine and suicide genes to enhance the activity and safety of tumor-specific cytotoxic T lymphocytes. Blood. (2007) 110:2793–802. doi: 10.1182/blood-2007-02-072843, PMID: 17638856 PMC2018664

[77] Van den Eynde A Gehrcken L Verhezen T Lau HW Hermans C Lambrechts H . IL-15-secreting CAR natural killer cells directed toward the pan-cancer target CD70 eliminate both cancer cells and cancer-associated fibroblasts. J Hematol Oncol. (2024) 17:8. doi: 10.1186/s13045-024-01525-w, PMID: 38331849 PMC10854128

