# Correction: Perspective: IL-15 cytokine-armored NK cells as ready-to-use immunotherapy for diverse malignancies: therapeutic potential and toxicity risks

**DOI:** 10.3389/fimmu.2026.1779800

**Published:** 2026-01-15

**Authors:** Scott L. Baughan, Timothy Folsom, Matt Johnson, Joshua Kreuger, Beau R. Webber, Branden S. Moriarity

**Affiliations:** 1 Department of Medicine, University of Minnesota Twin Cities, Minneapolis, MN, United States; 2 Center for Genome Engineering, University of Minnesota, Minneapolis, MN, United States; 3 Department of Pediatrics, University of Minnesota, Minneapolis, MN, United States; 4 Masonic Cancer Center, University of Minnesota, Minneapolis, MN, United States

**Keywords:** natural killer (NK) cells, chimeric antigen receptors (CARs), CAR-NK cells, IL-15 cytokine-armored CAR-NK, cytokine release syndrome, CAR-T cell therapy, cytokine release syndrome (CRS)

The following **Funding** information was erroneously omitted: “Catamaran Bio, NIH grants R21CA237789, R21AI163731, P01CA254849, P50CA136393, R01AI146009, U54CA268069, Children’s Cancer Research Fund, Cure Childhood Cancer, the Randy Shaver Cancer Research and Community Fund to Beau R. Webber and Brandon S. Moriarity, and from Office of Discovery and Translation, NIH grants (R01AI146009, R01AI161017, P01CA254849, P50CA136393, U24OD026641, U54CA232561, P30CA077598, U54CA268069, DOD grants HT9425-24-1-1005, HT9425-24-1-1002, HT9425-24-1-0231), and Children’s Cancer Research Fund, the Fanconi Anemia Research Fund, and the Randy Shaver Cancer and Community Fund to Brandon S. Moriarity”.

The original version of this article has been updated.

